# Inertial control: a novel technique for in-vitro analysis of foot function

**DOI:** 10.1186/1757-1146-5-S1-O42

**Published:** 2012-04-10

**Authors:** Tassos Natsakis, Koen Peeters, Fien Burg, Greta Dereymaeker, Jos Vander Sloten, Ilse Jonkers

**Affiliations:** 1 Mechanical Engineering Department, KU Leuven, Leuven, 3000, Belgium; 2 Department of Kinesiology, KU Leuven, Leuven, 3000, Belgium

## Background

In-vitro gait simulations have great potential, allowing a systematic analysis of the foot function. However, it is important that the loading conditions are realistic i.e. physiologic ground reaction forces (GRF). In most experiments, in-vivo measured GRF can be imposed [1,2]. However in experimental designs that evaluate the effect of altered muscle forces on foot motion this is more complex; the effect of the altered muscle activity on the loading and kinematics cannot be taken into consideration. Therefore, we investigated the use of a new technique to simulate such cases with realistic loading conditions.

## Methods

Our gait simulator simulates the activation of nine muscles (grouped in six groups), based on electromyography measurements. The forces are applied with pneumatic actuators and are measured by load-cells located between the tendons and the actuators. The set-up is able to simulate knee motion, using a motor for the horizontal and a platform under the foot for the vertical direction. The stance phase is simulated in 0.8 seconds.

The GRF in human gait is the sum of a static (human weight) and a dynamic part (acceleration of human mass). By applying a constant force on the platform (equal to the assumed weight of the subject), the measured GRF is the sum of the constant force and the force generated from the acceleration of the platform. This way, the kinetics are governed exclusively by the muscle activation.

## Results

**Figure 1:**
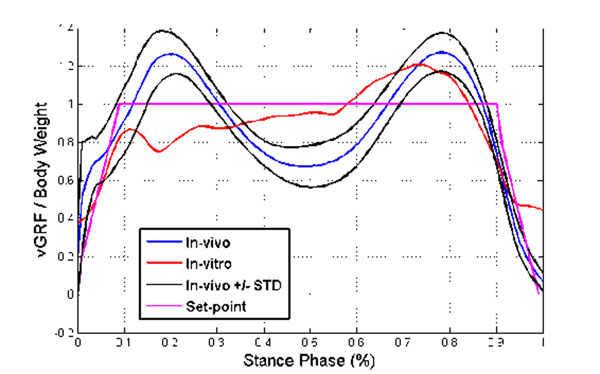
Comparison of the in-vivo and in-vitro measured GRF.

Four freshly frozen cadaveric specimens were used, for five measurements each. The in-vitro measured GRF was compared to in-vivo measurements (figure 1) and followed the normal pattern with an RMS error of 22%.

## Conclusions

Using this method, physiological GRF were reconstructed for normal gait, by reconstructing the mechanism that generates GRF. It could be, therefore, used for pathologic gait simulations, since the mechanism is identical.
